# A health promotion approach to emergency management: effective community engagement strategies from five cases

**DOI:** 10.1093/heapro/daab152

**Published:** 2021-12-12

**Authors:** J Hope Corbin, Ukam Ebe Oyene, Erma Manoncourt, Hans Onya, Metrine Kwamboka, Mary Amuyunzu-Nyamongo, Kristine Sørensen, Oliver Mweemba, Margaret M Barry, Davison Munodawafa, Yolanda V Bayugo, Qudsia Huda, Tomas Moran, Semeeh Akinwale Omoleke, Dayo Spencer-Walters, Stephan Van den Broucke

**Affiliations:** 1 Department of Health and Community Studies, Western Washington University, Bellingham, WA, USA; 2 Country Readiness Strengthening Department, WHO Health Emergencies Programme, World Health Organization, Geneva, Switzerland; 3 School of Global Public Health, New York University, New York, NY, USA; 4 Paris School of International Affairs, Sciences Po, Paris, France; 5 Department of Public Health, University of Limpopo, Sovenga, South Africa; 6 African Institute for Health and Development, Nairobi, Kenya; 7 Global Health Literacy Academy, Risskov, Denmark; 8 Department of Health Promotion and Education, School of Public Health, University of Zambia, Lusaka, Zambia; 9 World Health Organization Collaborating Centre for Health Promotion Research, School of Health Sciences, National University of Ireland, Galway, Ireland; 10 Department of Community Medicine, Faculty of Medicine, Midlands State University, Gweru, Zimbabwe; 11 Global Health, Thammasat University, Bangkok, Thailand; 12 Country Readiness Strengthening Department , WHO Health Emergencies Programme, World Health Organization, Geneva, Switzerland; 13 Health Security and Preparedness Department, WHO Health Emergencies Programme, World Health Organization, Geneva, Switzerland; 14 Global Infectious Hazards Preparedness Department, WHO Health Emergencies Programme, World Health Organization, Geneva, Switzerland; 15 Field Presence Cluster, World Health Organization, Abuja, Nigeria; 16 Faculté de Psychologie et des Sciences de l'Education, Institut de Recherche en Sciences Psychologiques, Université catholique de Louvain, Louvain-la-Neuve, Belgium

**Keywords:** emergency response, emergency management, community engagement, COVID-19, disasters

## Abstract

Community engagement is crucial for controlling disease outbreak and mitigating natural and industrial disasters. The COVID-19 pandemic has reconfirmed the need to elevate community engagement to build equity, trust and sustained action in future health promotion preparedness strategies. Using the health promotion strategy of strengthening community action enhances the opportunity for better outcomes. There is, therefore, a need to improve our understanding of community engagement practices during crises, scale-up good community engagement initiatives, and improve and sustain people-centered approaches to emergency responses. This paper presents five case studies from the United States, Singapore, Sierra Leone, Kenya and South Africa that demonstrate the potential strengths that can be nurtured to build resilience in local communities to help mitigate the impact of disasters and emergencies. The case studies highlight the importance of co-developing relevant education and communication strategies, amplifying the role of community leaders, empowering community members to achieve shared goals, assessing and adapting to changing contexts, pre-planning and readiness for future emergencies and acknowledgement of historic context.

## INTRODUCTION

The outbreak of COVID-19 in 2020 has shown the world that globalization, international travel, migration, interdependency of sectors, globally interconnected production and supply chains, and intense social exchange make its population vulnerable to crises of all types (Omoleke *et al.*, 2016; Abubakar *et al.*, 2018). While challenging the sustainability of the healthcare services and exposing the limitations of public health systems already reduced in capacity as a result of years of austerity policies, COVID-19 sent a shockwave through all sectors of society (Dixon *et al.*, 2020; Ramirez-Valles *et al.*, 2020; Saboga-Nunes *et al.*, 2020; Sherpa, 2020). The COVID-19 pandemic has literally unmasked the unprepared systems across all sectors to respond to such crises. At the same time, the experience with COVID-19 made clear that biomedical interventions alone have only limited effectiveness and take time to develop. Even when treatment and vaccines become available, distribution and ensuring uptake remain complex. Hence, there is also a need for approaches that are social and behavioral in nature. Many of the measures that are implemented to contain the pandemic, such as physical distancing, face mask wearing, hand hygiene or vaccination require a change of behavior on the part of citizens and health workers. In addition, the public’s perception that existing health systems cannot guarantee the protection of all citizens, and the feeling that some measures to respond to the pandemic are unnecessary or unjust, create a need for people to regain control of their health and to deal with the disruptive consequences of the pandemic (Chan *et al.*, 2020).

The expertise to address these needs is available in health promotion. While previous research has shown the value of health promotion for emergency responses (Lhussier *et al.*, 2016), the COVID-19 pandemic has lent some urgency to further examine the contribution of health promotion in emergency situations and to draw important lessons for possible future crises. These lessons relate to various aspects of health promotion. For instance, according to Ramirez-Valles *et al.* (2020), the COVID-19 crisis reminds us that health behavior is embedded in politics, culture, and social–political systems, and that prevention must be guided by principles of social justice and ecological responsibility. Sentell *et al.* and Abel and McQueen, on the other hand, highlight the importance of individual, community and population health literacy (Abel and McQueen, 2020; Sentell *et al.*, 2020). Studies have also indicated the importance of effective health education aimed at increasing knowledge and understanding—increasing knowledge and understanding of disease transmission and health promoting behaviors that support infection prevention and control (Wang *et al.*, 2018). The experience with communication in times of disasters shows that information should be adapted to the literacy needs of the people it intends to reach, with special attention for those who are the most vulnerable in pandemics, such as older people, migrants or people with disabilities, to allow timely and appropriate action in emergency situations (Smith and Judd, 2020; Sørensen, 2020). COVID-19 has also shown that in the current information society a pandemic is often accompanied by an ‘infodemic’, i.e. the overwhelming, rapid and far reaching spread of (sometimes incorrect) information, which means that addressing a health crisis also involves dealing with inadequate or misleading information. During a pandemic, this overabundance of information—some accurate and some not—can lead to confusion and ultimately mistrust in governments and the public health response (Limaye *et al.*, 2020). Van den Broucke points out that health promotion must seek to understand the unique circumstances of an emergency situation and take account of the way decisions are made during the crisis, how institutions respond to them, and how communication is impacted (Van den Broucke, 2020). To learn about the virus and ways to protect themselves, people actively select information sources and information from within these sources, some of which may be contradictory. The selection of information sources and the activation of cognitive schemes to filter, classify and assimilate information and judge the importance of possible measures can cause various biases, such as negative information bias (i.e. the tendency to attach more importance to negative than to positive information), positive information bias (i.e. the tendency to consider oneself as less at risk for negative consequence) or familiarity or recency bias (i.e. considering things that are familiar or recent as more ‘true’ as they can more easily be retrieved from memory). The fact that information about COVID-19 is widely diffused via social media enhances the risk of false information being accessed and being reinforced by the ‘echo chamber’ or ‘illusion of truth’ effect. Experience with previous pandemics may offer learnings on how to counter these effects by applying basic principles of information provision. Such learning is urgent, as several environmental and social transitions increase the likelihood of more frequent crises in the future (WHO, 2018).

Another key strategy for effectively responding to emergencies is to meaningfully engage communities in the response and management of the emergency. Effective responses to disease outbreaks require more than just information dissemination about a disease and the ways to prevent it. Consistent with health promotion’s focus and understanding of the social determinants of health, it is necessary to unpack the social context in which decisions are made and protective actions taken (Cialdini, 2007; Ariely, 2008; UN, 2011; Datta and Mullainathan, 2012; Waisbord, 2014; Haider, 2017). As people’s ability and willingness to individually and/or collectively adopt protective behaviors are shaped by an interplay of determinants at the individual, family, community, policy and societal level, the interdependence and interrelatedness of these different factors needs to be considered. Rather than directing the information to the ‘general public’, engaging members of communities in efforts to address the crisis can strengthen their capacity to deal with the disruptive effects of a pandemic at organizational and community level, and as such make a substantial difference in health outcomes.

This paper will briefly summarize the evidence guiding current community engagement strategies, share a selection of positive case examples, consider these strategies in relation to health equity, and raise questions on emerging and contentious issues for the field.

## THE NEED FOR COMMUNITY ENGAGEMENT IN EMERGENCY SITUATIONS

The World Health Organization’s (WHO) legal definition of a public health emergency of international concern is ‘an extraordinary event that may constitute a public health risk to other countries through international spread of disease and may require an international coordinated response (WHO, 2005, p. 17).’ Nelson and colleagues propose a definition of emergency preparedness that speaks more directly to local responses: ‘public health emergency preparedness (PHEP) is the capability of the public health and healthcare systems, communities, and individuals, to prevent, protect against, quickly respond to, and recover from health emergencies, particularly those whose scale, timing, or unpredictability threatens to overwhelm routine capabilities. Preparedness involves a coordinated and continuous process of planning and implementation that relies on measuring performance and taking corrective action’.

Many lives can be saved in the first hours and days of an emergency through an effective local response. The local population also plays the lead role in recovery and reconstruction efforts. Therefore, the capacity of a community to respond to a crisis, its activities in terms of primary healthcare, and the roles of local health workers, civil society and the private sector are central to effective emergency management (WHO, 2019). Health promotion, by centering participation, empowerment and community action in education, communication and skills development, can enable people to gain more control over their health in the context of emergency response and management (WHO, 1986; Laverack, 2017).

Previous epidemics and pandemics like Ebola, SARS, H1N1 and Zika have demonstrated the importance of community engagement in times of public health emergencies (Laverack and Manoncourt, 2016; Toppenberg-Pejcic *et al.*, 2019; Gilmore *et al.*, 2020; Maher and Murphet, 2020). Based on these experiences, the World Health Organization in January 2020 issued guidelines for implementing Community Engagement (CE) in response to COVID-19, recognizing it to be a necessary strategy for protecting vulnerable communities (Hu and Qiu, 2020; Wieland *et al.*, 2020). Specifically, WHO’s operational guidance for whole of society engagement alludes to the fact that community support is vital for critical functions, such as risk communication, case finding, contact tracing, cooperation with public health and social measures, and continuing primary healthcare (WHO, 2021). Communities and populations must be empowered to ensure that services and assistance are planned and adapted based on their feedback and local contexts.

Community engagement is critical because, as Laverack (2017) points out, ‘… disease outbreaks can only be fully addressed by helping people to empower themselves, rather than by simply trying to change their behavior’ (p. 2). To achieve this, health communication messages always need to be relevant to context. Like all communication, health communication has its roots in culture and should, therefore, be tailored to the needs of the population concerned (Airhihenbuwa *et al.*, 2020). If not, well intended health messages might be ignored or refused because the source of the information is perceived as untrustworthy, because the content of the information is irrelevant to the community’s knowledge systems, or because the cultural or community context was not considered (IDS, 2015; Omoleke *et al.*, 2016). The health promotion literature gives several examples of such ineffective communication. Chilisa (2005), for instance, wrote about how health communication messages aimed at preventing HIV infection were dismissed by local communities in Botswana because they were irrelevant to the local understandings of disease processes. Problems with the acceptance at local level of health communication messaging were also encountered during the 2014 Ebola epidemic in Guinea, Liberia and Sierra Leone, where a lack of bottom-up communication, distrust, rumors, misinformation and service delivery failures contributed to the continued engagement in unsafe traditional practices (Laverack and Manoncourt, 2016; Omoleke *et al.*, 2016).

In both of these examples, the term ‘community’ refers to groups of people who share a common geographical locality as well as a common culture and shared beliefs and norms. But a community need not necessarily refer to a locality; it can also be simply defined in terms of a network of people tied together by a shared identity and a shared set of norms, without necessarily residing in the same place (Bradshaw, 2008). In responding to the COVID-19 pandemic, the concept of community engagement has been expanded to include ‘virtual’ communities and digital engagement, as much of the public discourse about the coronavirus is happening on social media (Ali, 2020). As a result, the sharing of information about COVID-19 within communities is not only affected by rumors, distrust and cultural incompatibility as experienced in earlier epidemics, but is also hampered by the incomplete, incorrect and sometimes intentionally misleading disinformation that is disseminated via the internet, the ‘dark web’ and different social media, creating confusion and feeding conspiracy theories (Cuan-Baltazar *et al.*, 2020; Romer and Jamieson, 2020; Tasnim *et al.*, 2020; Uscinski *et al.*, 2020). A novel and unique challenge to health communication efforts in this regard are the ‘opinion leaders’ on social media, who have a huge impact on the perceptions and beliefs of people in the community without even being in physical contact with them.

To mount an effective response to these challenges, efforts to inform and encourage the population to adopt protective behaviors against the pandemic can benefit from combining advice from experts with local community knowledge and leadership. Community leaders and members should be engaged throughout the planning, implementation and evaluation of interventions in a manner that seeks to understand local perspectives, solicits input, shares information and collaboratively addresses the outbreak (Airhihenbuwa *et al.*, 2020; WHO, 2017, 2020). Comparable coordination mechanisms should be employed or established at other levels of government and among different levels of government. Local authorities and community groups can reduce the risks faced by the local community and should be ready to address the needs of affected populations requiring assistance, including people with higher levels of social, economic and health vulnerability. Hence, local governments should coordinate planning and action with local agencies, local or district offices of ministries, and civil society organizations, and with other levels of government (WHO, 2021).

Another reason why community engagement is important is that emergency situations have a magnifying effect on existing problems. The impact of the Ebola outbreak in West Africa in 2014–2015, for instance, was complicated by a fragile healthcare infrastructure (Laverack and Manoncourt, 2016). In a similar vein, COVID-19 has been a catalyst for exacerbating preexisting inequities in society, as shown by the higher rates of infection, morbidity and mortality among vulnerable populations like the Black, Indigenous, communities of color, people experiencing homelessness or living in crowded conditions, low income communities, and incarcerated people (Alberti *et al.*, 2020; Leitch *et al.*, 2021). This magnifying effect may well be related to the issue of trust: the existing inequities and the historical and structural processes that led to the disenfranchisement of these vulnerable groups help explain their distrust and skepticism toward health advice, and toward the health system in general (Leitch *et al.*, 2021). Legacies of colonialism and medical experimentation linger in many settings and profoundly impact the beliefs and willingness to engage in recommended behaviors (Miller, 2007; Mosby and Swidrovich, 2021; Tilley, 2020).

On the other hand, COVID-19 has also highlighted that impoverished and vulnerable communities are not mere *sources* of problematic responses to disease threat, but are crucial *re*sources for pandemic global preparedness and response, through various forms of community engagement (Leach *et al.*, 2021). The response to the Nepal earthquake and similar experiences made clear that community engagement can facilitate a more efficient use of resources, strengthen coordination and build local capacities (UN OCHA, 2016). In this regard, the call to include hard to reach populations, women leaders, people with disabilities, and minorities in preparedness and response planning is getting louder. Understanding their perspectives and including them in intervention planning and delivery is critical to addressing effectively the complexity and diversity of health emergencies, disasters and humanitarian crises (De Weger *et al.*, 2020; Elisala *et al.*, 2020; Patel *et al.*, 2020).

## METHODS AND STRATEGIES FOR COMMUNITY ENGAGEMENT IN EMERGENCY SITUATIONS

While it is increasingly recognized that communities must be engaged throughout the full cycle of emergency preparedness, readiness, response and recovery, the question remains *how* community engagement can be ensured. Strategies to strengthen community engagement and to adapt evidence-based practice to local needs and circumstances are well-known to health promoters, but emergency situations can complicate the implementation of these strategies. With regard to COVID-19, for instance, the mechanisms that are typically used for building community engagement, such as organizing community meetings, have proven difficult to roll out when quarantine measures and lockdowns are limiting the possibility of meeting others (Tindana *et al.*, 2020). Moreover, in crisis situations community engagement can be complicated by fear (Shrivastava and Shrivastava, 2020). An illustration of this is given in Zhu and Cai (2020), as they describe the disorderly reactions of the residents of Wuhan in the early part of the lockdown. Fear can also be a driver of stigma and discrimination. Several studies conducted through the course of the pandemic response have revealed discrimination against health workers in some countries as well as against vulnerable populations such as refugees and migrant workers (RCCE Collective Service, 2021). The imperative to take swift action during a crisis can also be problematic for community engagement. It is not evident for communication to be at the same time engaging communities and enabling swift and nimble behavior change, except perhaps in the case of a community-initiated response (Artelle *et al.*, 2020). While social media can be a useful resource for quickly disseminating information about risks and protective actions to a large audience, it also carries the risk of information errors and misinformation (Ekzayez *et al.*, 2020), and depends on development level in a particular setting such as the local internet coverage.

There is, therefore, a need to improve our understanding of community engagement practices during crisis, scale-up good community engagement initiatives, and improve and sustain people-centered approaches to emergency responses (IFRC, 2019; Bardosh *et al.*, 2020; UNICEF, 2020). Some of this understanding is beginning to emerge from the literature. For instance, Gonah (2020) notes that effective community engagement during crises relies on partnership and collaboration with relevant groups, clear plans and guidelines, well-established coordination structures, and transparent reporting and documentation. Gilmore *et al.* (2020), in their rapid review of the gray literature on community engagement during communicable epidemics, identified six categories of actors who are vital for successful community engagement efforts in crises: local leaders, community and faith-based organizations, various community groups, health system committees, individual community members and key stakeholders. Governments need to involve civil society organizations, local communities and the local workforce in developing plans for managing the risks and impacts of emergencies and disasters. They also identified key activities that should be supported during intervention implementation to include these actors: planning and design, introductions and trust building, developing communication strategies, surveillance and tracing, logistics and administrative tasks. Toppenberg-Pejcic *et al.* (2019) performed a review of the literature on emergency risk communication strategies in response to Ebola, Zika, and Yellow Fever, and concluded that communities should be engaged in developing their own emergency risk strategies well before the next crisis, so the structures are ready to be activated with haste (Bedson *et al.*, 2020).

These studies help to identify the conditions, strategies and actors for enhancing community engagement in the context of responding to a health crisis situation. However, they remain rather descriptive in nature, which reduces the replicability of their findings. Moreover, the conclusions and lessons they draw are rather general and not embedded in a theoretical framework. This study aims to add to the existing understanding of community engagement in the context of addressing emergency situations by presenting five case studies and analyzing the strategies used to develop or enhance community engagement in a systematic way, in accordance with health promotion principles (Laverack, 2017).

## METHOD

### Case data sources

The cases presented in this study come from two sources. Three cases were drawn from the World Health Organization (WHO) Community Engagement Package database. This database serves as a repository of experiences of community engagement practices in different settings, contexts and groups. It showcases good practices, lessons learned, challenges, and innovative approaches to working with communities in WHO regions. It is also used for developing learning resources on community engagement. We selected three cases from among 52 in the health emergencies and disaster management category from this database, based on the following inclusion criteria: (i) Case studies documenting good health promotion principles and strategies, with innovations and potential to adapt and scale up across regions in emergency context; (ii) Cases representing different emergency settings; (iii) Cases reflecting COVID-19 and other health emergencies; and (iv) Cases published within the last 10 years.

Cases 4 and 5 are drawn from the IUHPE COVID-19 Response for African Region project, focusing on implemented actions in communities in Kenya and South Africa. The project aimed to engage with key partners in Africa to plan and implement a range of risk communication and community engagement measures, based on health promotion principles, to stop the spread of COVID-19 within communities, equitably, while protecting people’s basic needs and their physical and mental health. Case studies from two of the four participating countries in this project are included in this paper, one from South Africa and one from Kenya, where community engagement activities were implemented with disadvantaged communities, including black townships and informal settlements, in collaboration with local agencies, partners and networks.

### Analysis

Two matrixes were created as analytical tools for data analysis. One was informed by social–ecological theory (Bronfenbrenner, 1979, 2005; Glanz *et al.*, 2008) and was designed to capture key details about the context of the intervention. The second matrix was developed from the Bergen Model of Collaborative Functioning (Corbin and Mittelmark, 2008) to ascertain information on the process of implementation with special attention to collaboration among stakeholders. For Cases 1–3, the WHO team analyzed texts to complete the raw matrixes. For Cases 4 and 5, country leads responded to questions based on these matrixes. These qualitative data were then examined for key themes and presented in this report accordingly.

## RESULTS

### Case study 1: Engaging the community in a chemical disaster recovery, Graniteville, USA

#### Background

The source of information for this case, included in the WHO database, was published in the International Journal for Environmental Research and Public Health (Abara *et al.*, 2014). The town of Graniteville, South Carolina, suffered a chlorine spill which led to deaths and injuries requiring medical attention due to inhalation of chlorine (Abara *et al.*, 2014). Whereas past disaster responses did not adequately address community engagement, the chlorine disaster response in Graniteville was different, as the research team from the University of South Carolina, Columbia, from the onset engaged key stakeholders from academic, civil society, private organizations and residents in the response efforts. To that effect, the research team partnered with local stakeholders to identify and address locally identified health and environmental concerns. Through these efforts, research and health services responded to and addressed community-identified concerns (Abara *et al.*, 2014).

#### Critical success factors

Trust was crucial and the engagement through town hall meetings facilitated the building of relationships between the community and the external responders. The community advisory board fostered local ownership that catalyzed local resource mobilization to support the response efforts. The recruitment and involvement of community members also empowered them and increased their sense of agency (Abara *et al.*, 2014). The community’s early engagement was crucial and contributed to the initiative and partnership’s success by providing additional resources and navigating technical and managerial capacity gaps. According to the authors, it is relevant to engage and mobilize communities faced with different types of disasters, which allows to adopt multisectoral, speedy and inclusive response approaches to get the job well done. Also, listening to affected people is essential in planning successfully for emergencies (Abara *et al.*, 2014).

#### Key challenges

The authors indicated that past disaster responses did not adequately address community engagement. The chlorine disaster response in Graniteville benefited from the support of the research team from the University of South Carolina in addressing the CE issue. However, it is not indicated if the Public Health authorities in South Carolina will be scaling up the community engagement intervention in preparedness, response, and rehabilitation or recovery phases based on lessons learnt in Graniteville and adapted to the realities in other parts of the state.

### Case study 2: Community-Led Ebola Management and Eradication in Sierra Leone

#### Background

The source of information for this case, taken from the WHO database, is the Community-Led Ebola Management and Eradication (CLEME) program report prepared by Action Contre la Faim International (ACF, 2015). This NGO partnered with District Health teams in Sierra Leone to introduce the CLEME approach. During the 2014–2015 Ebola outbreak, ACF collaborated with health authorities and district partners in Moyamba and Kambia. The report describes the rapid early spread of Ebola because communities did not fully adhere to the recommended safety measures, which was attributed to weak social mobilization and community engagement practices (ACF, 2015). The ACF’s CLEME approach employed a five-phase process that included selecting communities and assessing them on the Ebola outbreak and dangers; applying a Participatory Rural Appraisal (PRA) methodology; developing a community-guided action plan; and ensuring long-term safety by following up on the CLEME approach (ACF, 2015).

#### Critical success factors and core health promotion principles

The program involved community members in multiple stages of the CLEME process to build trust and relationships. The response teams used communication and engagement strategies that enhanced community members’ participation and addressed the needs of low-literate members of the community. Many of the small-sized communities that implemented CLEME had higher group participation. As a result, the program hastened social cohesion and built ownership of the community action (ACF, 2015).

According to the report, the program’s success factors included a continuous analysis of the situation and adaptation of messages and strategies to align with the prevailing situation context. It also incorporated tools and strategies that addressed women’s, men’s, boys’ and girls’ unique needs. Follow-up visits to the communities and constant reviews helped the continuous improvement of the engagement process. Equally important was the integration with existing community-based initiatives and programs, which was critical for the community engagement efforts and the systems’ viability and continuity of community mobilization and involvement in emergencies (ACF, 2015).

#### Key challenges

The NGO collaborated with the health partners to plan and roll out the community engagement intervention during the 2014–2015 Ebola outbreaks. However, it was uncertain that the project would be scaled up and continued after the funding lapses at the end of the outbreak and mainstreamed in the national response and health promotion framework. Also, the implementation of the CLEME project was quite successful in small rural communities and not quite adapted to the larger and heterogynous populations in the urban settings.

### Case study 3: Community engagement for migrant workers’ response to COVID-19 in Singapore

#### Background

The source of information for this case, maintained in the WHO database, is the Wai Jia Tam’s report (2021) on the Migrant Workers Risk Communication & Community Engagement Project, by My Brother SG and the National University of Singapore. Migrant workers, mainly from Bangladesh, India and China, make up a sizable percentage of the Singapore population. They often live in highly overcrowded dormitories with an occupancy rate that far exceeds what is approved by authorities, which makes them vulnerable to COVID-19 infection (Tam, 2021).

According to the report published in August 2020, a local NGO (My Brother SG) and the National University of Singapore initiated a Risk Communication and Community Engagement project to address the specific communication and engagement needs of this migrant worker population. This was motivated by the high number of confirmed cases of COVID-19 among migrant workers residing in crowded dormitories. At the beginning of the RCCE project, an assessment of communication requirements was done, followed by developing and adapting information products, and recruiting and forming teams. The project trained team members, built synergies with government and other agencies, set up platforms and held coordination and resource mobilization meetings.

The report indicated that public health measures were taken regarding workers that tested positive and followed up the contacts. Team members took steps to ensure social distancing. Culturally sensitive communication and engagement strategies were deployed, and engaged workers practiced the recommended behaviors to keep workers safe from COVID-19 while quarantined. The report also highlighted a collaboration with relevant government agencies and local organizations to develop information products through appropriate formats and channels. Furthermore, the project teams adopted strategies that resonated with specific target audiences, such as understanding hierarchies, co-creating products, organizing participatory workshops and incorporating community feedback in decision making. Efforts were made to enhance two-way communication between external responders and the affected migrant communities.

#### Critical success factors

The report revealed that the strategies that were adopted reinforced trust and relationships, and forged connections with migrant workers’ communities. The participatory approaches such as storytelling theater and film discussions created opportunities for reinforcing relational connections and the process built in the entire engagement contributed to the project's stakeholder participation and eventual success. Another successful strategy involved inviting migrant worker social media influencers to co-host regular webinars on their online platforms to promote dialogue, address concerns and promote trusted health advice. Listening to the affected people and establishing two-way communication and co-creation addressed their concerns, which enabled the incorporation of their perspectives in the project design and rollout. The project's adoption of a people-centered strategy prioritized a bottom-up participatory approach and included a wide range of stakeholders, which helped reduce negative consequences that characterized communication in the early phase of the COVID-19 response.

#### Key challenges

As human and material resources and systems for coordinating stakeholders were not put in place in the preparedness and readiness phase, this might have contributed to the initial weak response to the COVID-19 pandemic. It is necessary to build a relationship early with the affected population in order to enhance their sense of empowerment and ownership.

### Case study 4: COVID-19 response for the African Region project in Kenya

#### Background

This project was undertaken by the African Institute for Health and Development (AIHD) and IUHPE/Vital Strategies, in close collaboration with the Nairobi Metropolitan Services (NMS), the Ministry of Health (MoH) at the National and County levels, and key stakeholders. The health promotion project was implemented in the Ruai and Njiru wards of the Kasarani Sub-County in Nairobi County (IUHPE, 2021). It used the community engagement strategy to engage the NMS and key partners in the COVID-19 response. Working in collaboration with national and local agencies and partners, Health Promotion Officers (HPOs) were employed to implement intervention activities at the community level and help train the Community Own Resource Persons (CORPs) that included religious and traditional leaders, community health workers and volunteers, women and youth leaders in the project area.

The intervention areas presented unique characteristics. Ruai ward hosts among other groups, the Maasai community, whose traditions and culture require them to live a nomadic lifestyle while living within closed communities. The ward also experiences challenges of access to water, sanitation and hygiene (WASH). The Njiru ward, on the other hand, is characterized by a high-density population of young people, an extremely busy transport and market sectors. It is home to a busy *matatu* (a mini bus or similar vehicle used for transport) terminus and is characterized by cultural diversity in terms of religion, ethnicity and social status. According to the 2019 population census, the Kasarani Sub-County has a total population of 398 902 people, with 94 499 dwellers in Njiru and 105 485 in Ruai (Kenya National Bureau of Statistics, 2019).

The selection of CORPs was conducted through consultation between the Sub-County Health Promotion Community Services team leads and the targeted risk groups. The CORPs were selected based on their level of positive influence, acceptability and the respect they command within their groups. Influencers were drawn from the various risk groups, including the youth, religious leaders, *bodaboda (motorcycle)* riders, *Matatu* crew, market vendors and the Maasai community. The CORPS denoted community involvement while executing implementation activities aimed to encourage community entry, acceptance, ownership and sustainability of adoption and promotion of positive social behavior practice during and beyond the project phase. The identified CORPs received training, were commissioned as change agents and encouraged to continue community conversations at individual, family, community, and workplace levels and within other social spaces. They were equipped with information and skills that would allow them to provide correct information, clarify myths, challenge misconceptions and misinformation on COVID-19, and address other health-related concerns.

#### Critical success factors

The project organization structure, which involved global and local health promotion experts, enhanced project inception and implementation. An additional strength of the project was the partnership between the MoH, NMS and the AIHD, who shared the common goal of engaging the community in the COVID-19 response. The support of the county and sub-county administrative leaders provided an additional layer of support for the team.

A key success factor was the intervention’s multi-strategy approach through advocacy, community engagement, social mobilization and media communication. Advocacy ensured smooth community entry into the project area, capacity building of the CORPs resulted in a shared understanding of the strategies, approaches and activities, and the communication strategy ensured that the key messages were tailored to the needs of local communities. The use of Community Engagement Forums addressed the misinformation, myths, rumors and misconception in the community while imparting the right information. The use of media (a local radio station and public address system) as a key communication strategy enhanced information dissemination to the target population by HPOs. Awareness creation through transect walks and public address systems with branding proved successful, as it created curiosity, thereby increasing the reach. The CORPs and facilitators donned branded reflector jackets and sashes with a unifying message at the front and back sides stating ‘*#Social Mobilizers #Change Agents*’. Testimonials from COVID-19 survivors enabled the community to understand the disease through lived experience.

The project incorporated the deaf and physically disabled as CORPs to disseminate information to their social groups, this fostered inclusion and acceptability by their social groups.

#### Key challenges

The COVID-19 health promotion activities were initially coordinated by the MoH at the national level, with limited attention to the decentralized and community structures. Consequently, some of the decisions taken did not take the individuals and their communities’ circumstances and contexts into consideration (for instance, guidance on social distancing in crowded poor informal communities). Consultations with community leaders and opinion leaders were at most *ad hoc*, while the use of the community health structures was limited. Lack of trust in the Government plays a key role in the way people respond to the health directives.

On the other hand, the study results show the importance of working with local people to sensitize communities on COVID-19 and the utility of employing accessible media. This requires governments to invest in RCCE as a key strategy for preventing and controlling the pandemic, while putting measures in place to address the physical barriers to prevention, including providing access to water in areas where this is a challenge.

### Case study 5: IUHPE COVID-19 response for African Region project: South Africa

#### Background

This intervention by IUHPE/Vital Strategies as a complement to the efforts of the national Department of Health and key NGOs and CBOs responding to the COVID-19 pandemic focused on resource-limited rural and urban settings within three districts (Waterberg, Vhembe and Sekhukhune) in the Limpopo Province of South Africa (IUHPE, 2021). These locations are mining towns surrounding communities with poor access to COVID-19 diagnosis and healthcare facilities. Given that the main aim of the intervention was to enable members of the community to take behavioral actions to prevent COVID-19 infection, emphasis was placed on decision-makers within households.

The three districts were chosen on account of their high COVID-19 prevalence rates. Since this project involved well-developed health promotion strategies, it focused on more remote communities where the interventions from the government were not as present. The work began with translating risk communication messages into local languages and engaging local people to bring those messages to community members. The team collected baseline data and conducted intervention activities, which included producing an app for household preparedness (which was originally designed by DOH but was not successfully rolled it out in these communities). A total of 8000 community health workers (CHW) were identified and supplied with mobile phone on which an app was installed, connected to the DOH. The team also conducted a ‘Train the Trainers’ workshop to enable CHWs to support their colleagues in using the app.

Additionally, efforts were made to tap into existing networks, including traditional leaders, healers, religious leaders, artists, teachers and school staff. This group formed a coalition made up of appointed coordinators, which was supplied with personal protective equipment (PPE) and informational materials customized to the local language. They used SMS messages to communicate on mobile devices and local radio to send health messages. Following the interventions, data were collected to trace the effectiveness of the strategy in terms of gains in knowledge, changes in the attitude of the community toward the pandemic, practice of protective behavior (handwashing, wearing face masks, etc.), and the people’s ability to recognize and address misconceptions and misinformation.

#### Critical success factors

A key success factor was the partnership with the DOH and the University of Limpopo and the Education Center. The local partnership ensured a high level of trust and very good cooperation. The translation of risk communication messages into local languages worked to dispel misconceptions. When the intervention community had a spike in cases, the community members responded and were able to quickly reverse the trend. When a new variant of the virus emerged, the training of CHW helped to change attitudes and behavior within the community. An additional strength was the organization of the project, which provided a useful cross-circulation of ideas through the international collaboration. The partnership between global and local health promotion experts within the various countries supported the implementation of robust health promotion strategies, while the materials, frameworks and documents shared by the project lead provided useful and relevant support throughout the project.

#### Key challenges

A number of structural factors were identified as prohibiting those who may wish to follow the preventive measures, and this ultimately affected the expected success of the intervention strategies. Such factors include the following: inadequate water supply; housing that does not allow for social distancing; low levels of health literacy; and unfavorable socio-cultural practices. Economic factors also played a significant unfavorable role. The fact that most people earn less than USD 10 per month in the villages certainly made it difficult for them to make ends meet, let alone buying soap or sanitizer for hand hygiene, for example. In addition, the Government relief package, meant to support citizens in need, fell short of achieving its objectives.

It was also evident from the evaluation data that people had a lot of misinformation and misconceptions at baseline. Although there was a positive significant shift in knowledge of community members and positive change in perception regarding all the myths and misconceptions/misinformation measured at follow-up, these changes were minimally translated into practice. Strategies such as RCCE that apply health promotion principles and methods, such as developing personal skills to make informed behavior change decisions and maintain them, is key in the fight against COVID-19. These strategies were limited and any existing ones are at their infancy. Creating a supportive environment will significantly go a long way in enabling people to translate knowledge into action that will maximize positive behavior changes conducive to curbing the spread of coronavirus within rural communities in Africa.

## DISCUSSION

Community engagement is crucial for controlling disease outbreak and mitigating natural and industrial disasters. The COVID-19 pandemic has reconfirmed the need to elevate community engagement and health literacy to build equity, trust and sustained action in future health promotion preparedness strategies (Levin-Zamir *et al.*, 2021). Only community-led and people-centered approaches can combine the complementary strengths of a broad set of partners to support and leverage the widest possible uptake, which is so important for an effective epidemic response. As global and regional efforts are being harnessed toward more people-centered, community-led and whole society approaches to addressing pandemic emergencies (IFRC, 2019; WHO, 2020), platforms such as the RCCE Collective Service, a new partnership between the WHO, UNICEF and the IFRC with active support from the Global Outbreak Alert and Response Network (GOARN), can strengthen the coordination and practice of community engagement and foster a more collaborative COVID-19 response. Yet as the need for community engagement to respond to a health crisis situation is increasingly acknowledged, more information is needed about the key conditions, strategies and critical success factors that can sustain community engagement in the context of a health crisis. Such information can be derived from concrete examples.

The cases from USA, Sierra Leone, Singapore, Kenya and South Africa presented in this paper demonstrate several key features of best practices in community engagement, drawing on health promotion principles and methods. In line with WHO guidance (WHO, 2020), future emergency responses must involve the community members for project ownership and sustainability. As adequately documented by the cases from USA and Singapore, they must allow for community members’ agency and active involvement. These efforts should build on existing assets and make use of ongoing relationships, local expertise, existing infrastructure and community leaders. The best responses combine diverse forms of expertise: cultural, technical, logistical and relational. Ideally, community engagement should also happen early. The best way to achieve that, as seen in the case examples, is to build and maintain community engagement and emergency infrastructure before disaster hits.

### Relevant education and communication strategies

Successful implementation strategies, as evidenced in the cases presented in this article, can involve a range of practices grounded in a health promotion approach. The translation of risk communication materials into appropriate languages is only a starting point. Ensuring that the messages are also accessible to non-reading community members through diverse media is also crucial. The incorporation of artistic modes of dissemination enables co-creation, relevance and uptake within communities (Corbin *et al.*, 2021). The Singapore Kenyan and South Africa cases above demonstrate the utility of theater, roleplay, storytelling, film, music, text, local radio and other creative communication channels as helpful for conveying information, engaging in two-way dialogue, and building trust and relationships.

### Community leaders

Another important aspect is who delivered the messages and/or programming. In the Singapore case, social media influencers from within the migrant worker community helped to elevate discussions of COVID-19 prevention and mitigation strategies. In the Kenya case, community walks with prominent community leaders and members clearly identified with branded vests and sashes amplified health messages and encouraged dialogue. They also meaningfully incorporated testimonials from a COVID-19 survivor to help expel fear, myths and misconceptions. The Kenya team also enlisted people with disabilities as change agents, for example by involving the deaf community as change agents to the hearing community and within their social groups.

### Shared goals

A re-occuring theme among the cases is the unifying power of a diverse group of stakeholders to address a common objective. Responding to emergencies might lend itself more easily to this kind of unified action, since communities are generally eager to resolve crises and to help. During the chlorine gas disaster, the ability of community members to serve in the recovery effort contributed to a sense of contribution and agency (Abara *et al.*, 2014). In the IUHPE COVID-19 Response for African Region collaboration in Kenya and South Africa, the global collaboration including the ability to share ideas with regional and international colleagues, as well as the sharing of materials, frameworks and documents supported the implementation of robust health promotion strategies.

### Adapting to changing context

Successful initiatives also engage processes for the continuous interrogation of the relevance of strategies for the evolving context. For instance, in the Sierra Leone case, the organizers engaged in the continuous analysis of the communities' barriers to safe behaviors so that messages and strategies can adapt to the evolving context. Adapting to the specific needs of vulnerable groups and sub-groups must be relentless, and community-led. In South Africa, because of heavy local involvement, when the new variant came, the program was able to quickly adapt and respond.

### Planning and readiness for future emergencies

A key success factor for strengthening community action in an emergency is the pre-existence of community relationships and infrastructure. Yet this requires a critical discussion of the challenges related to health promotion preparedness. For instance, is a health promotion preparedness system ‘a need to have’ or ‘nice to have’ (Levin-Zamir *et al.*, 2021)? The cases presented in this article provided multiple solutions to challenges as they appeared, but more research is needed to develop more readily available and sustainable solutions for the future, to identify the specific health promotion skills that are needed in emergency situations, and to specify how this capacity can be strengthened.

Moving beyond community mobilization and engagement, an effective strategy to combat disease outbreaks and address other complex emergencies, both natural and those created by humans, must factor in the concept of sustainability and therefore, a long-term goal of community resilience. Although initial attention is placed on responding to the various dimensions and evolution of an emergency and/or an outbreak, it is important to recognize that strengthening local capacity is critical so that communities have skills, competencies, and resources to address future, unknown challenges, such as climate change, natural disasters and those created by humans, civil conflict, etc. For example, in the IUHPE Africa COVID-19 Project, one of the key lessons learned in Kenya and South Africa was that much of the success of local activities was because they were designed to build on local strengths and resources, and implementation occurred through working in partnership with local communities and existing community structures. These enabled community residents to have a leadership role in informing their neighborhoods about COVID-19 and strengthened community members’ perceptions and ownership of the programme itself. By reinforcing community systems, local groups and social networks strengthen information dissemination, build relevant, trusted knowledge and focus skills and competencies that are needed to respond to the threat facing them.

### Acknowledgement of historic and current realities

There are also deeply embedded societal challenges to be acknowledged. As mentioned above, and seen clearly in the responses to Ebola and COVID-19, profound distrust in Western/Northern biomedical advice can be traced back to legacies of colonialism in many parts of the world (Miller, 2007; Tilley, 2020; Mosby and Swidrovich, 2021). As this distrust cannot be easily surmounted in emergency situations, addressing historic injustice needs to be a part of ongoing health promotion practice (Leitch *et al.*, 2021). Another societal challenge concerns the use of social media. While social media may offer many positive applications to deal with health emergencies, they also present certain challenges. Specifically, their role as a catalyst in enhancing the overwhelming, rapid and far-reaching spread of information, and especially in spreading and reinforcing inadequate or misleading information, means that addressing a health crisis also involves a need to develop critical health literacy (Abel and McQueen, 2020; Sentell *et al.*, 2020). Finally, broader societal issues such as nationalism, immigration, inequity and/or social isolation also threaten the response to global pandemics.

### Limitations

Despite best practices, the cases have specific weaknesses. The Singapore Migrant Project was implemented at a national level, and the rest of the cases described communication and community engagement experiences deployed in a few communities or locations at subnational levels. Nearly all the case studies were initiated during the response phase. However, it is better to build networks, leadership, communication and coordination structures, establish relationships and plan resource mobilization before an outbreak or disaster response and mainstream community engagement in the entire emergency management cycle. The Sierra Leone case study indicated the community engagement intervention thrived better in small rural communities. However, the whole of society approach must address more comprehensively urban and rural settings and appropriate legislation for broad participation and inclusiveness.

### Replicating successful initiatives in diverse settings

The successful strategies presented here might inform practices in other settings. Replicating these approaches require analyses of the community's governance and function: the socio-economic, political set-up, and operating environment; the scanning of existing public/community engagement approaches/tools, including platforms, communication formats, and trusted groups and channels and feedback mechanism. Once these analyses are done, it would be possible to implement strategies that fit in the new setting with any necessary adaptations.

## CONCLUSION

Emergencies are complex and turbulent with unpredictable impact. Protecting communities that are at risk from or affected by emergencies and disasters requires the participation of every member of those communities. Using the health promotion strategy of strengthening community action enhances the opportunity for better outcomes. Community-based organizations can adapt scientific and government messages and recommendations to achieve greater participation of populations and improve the effectiveness of public health and social measures. The cases presented in this paper demonstrate potential strengths that can be nurtured to build resilience in local communities to mitigate the impact of disasters and emergencies. The global scope of the paper shows that empowerment is an asset that can be applied across the world to harness people’s health and safety. Adapting health promotion approaches to local needs can enhance the communities’ power to act quickly when emergencies occur. The lessons learned illustrate the high capacity of people and communities to collaborate, communicate, and confront challenges despite being in vulnerable situations. However, there are also gaps in the extant literature, both with regard to documenting the process and outcomes of community engagement strategies in practice and in terms of the lack of evidence regarding the relative effectiveness of different approaches. Therefore, further empirical studies are needed, as well as the development of more integrative theoretical frameworks to guide health promotion practice in this important area.

## FUNDING

This research received no specific grant from any funding agency in the public, commercial, or not-for-profit sectors.

## DISCLAIMER

The authors alone are responsible for the views expressed in this article and they do not necessarily represent the views, decisions or policies of the institutions with which they are affiliated.

## Notes

Submission intended for the WHO Supplement for the 10th Global Conference on Health Promotion as an original submission.

## References

[daab152-B1] Abara W. , Wilson S., Vena J., Sanders L., Bevington T., Culley J. M. et al (2014) Engaging a chemical disaster community: lessons from Graniteville. International Journal of Environmental Research and Public Health, 11, 5684–5697. 24871259 10.3390/ijerph110605684 PMC4078542

[daab152-B2] Abel T. , McQueen D. (2020) Critical health literacy in pandemics: the special case of COVID-19. Health Promotion International. 10.1093/heapro/daaa141. PMC7799085 33351138

[daab152-B3] Abubakar I. , Aldridge R. W., Devakumar D., Orcutt M., Burns R., Barreto M. L. et al (2018) The UCL-Lancet commission on migration and health: the health of a world on the move. The Lancet Commissions, 392, 2606–2654. 10.1016/S0140-6736(18)32114-7 PMC7612863 30528486

[daab152-B4] ACF International. (2015) *Sierra Leone Case Study. Community-led Ebola management and eradication (CLEME): Trigger behavioural change to strengthen the community’s resilience to Ebola outbreaks*, 19 June. https://reliefweb.int/report/sierra-leone/acf-sierra-leone-case-study-community-led-ebola-management-and-eradication-cleme (last accessed 21 May 2021).

[daab152-B5] Airhihenbuwa C. , Iwelunmor J., Munodawafa D., Ford C., Oni T., Agyemang C. et al (2020) Culture matters in communicating the global response to COVID-19. Centers for Disease Control and Prevention, 17, E60. https://www.cdc.gov/pcd/issues/2020/20_0245.htm (last accessed 21 May 2021). 10.5888/pcd17.200245 PMC7367065 32644918

[daab152-B6] Alberti P. M. , Lantz P. M., Wilkins C. H. (2020) Equitable pandemic preparedness and rapid response: lessons from COVID-19 for pandemic health equity. Journal of Health Politics, Policy and Law, 45, 921–935. 10.1215/03616878-8641469 32464654

[daab152-B7] Ali S. (2020) Combatting against Covid-19 & misinformation: a systematic review. Human Arenas. 10.1007/s42087-020-00139-1.

[daab152-B8] Ariely D. (2008) Predictably irrational. Harper Collins, New York, NY.

[daab152-B9] Artelle K. A. , Brown K., Chan D. E., Silver J. J. (2020) A community-led approach to COVID-19. Science (New York, N.Y.), 369, 385–386. 10.1126/science.abd2107 32703870

[daab152-B10] Bardosh K. L. , de Vries D. H., Abramowitz S., Thorlie A., Cremers L., Kinsman J. et al (2020) Integrating the social sciences in epidemic preparedness and response: a strategic framework to strengthen capacities and improve Global Health security. Globalization and Health, 16, 120. 33380341 10.1186/s12992-020-00652-6 PMC7772799

[daab152-B11] Bedson J. , Jalloh M. F., Pedi D., Bah S., Owen K., Oniba A. et al (2020) Community engagement in outbreak response: lessons from the 2014-2016 Ebola outbreak in Sierra Leone. BMJ Global Health, 5, e002145. 10.1136/bmjgh-2019-002145 PMC7445350 32830128

[daab152-B12] Bhattacharyya S. , Lopes C. A., Nyamupachitu-Mago E., Gilmore B., Al E. (2020) Policy Brief: Community engagement for COVID-19 infection prevention and control: A rapid review of the evidence [Technical Report]. UNICEF. https://researchrepository.ucd.ie/handle/10197/11428 (last accessed 21 May 2021).

[daab152-B13] Bradshaw T. K. (2008) The post-place community: contributions to the debate about the definition of community. Community Development, 39, 5–16.

[daab152-B14] Bronfenbrenner U. (1979) The ecology of human development: experiments by nature and design. Harvard University Press, Cambridge, MA.

[daab152-B15] Bronfenbrenner U. (2005) Making human beings human: bioecological perspectives on human development. Sage Publications, Thousand Oaks, CA.

[daab152-B16] Chan H. F. , Brumpton M., Macintyre A., Arapoc J., Savage D. A., Skali A. et al (2020) How confidence in health care systems affects mobility and compliance during the COVID-19 pandemic. PLoS One, 15, e0240644. 33057450 10.1371/journal.pone.0240644 PMC7561184

[daab152-B17] Chilisa B. (2005) Educational research within postcolonial Africa: a critique of HIV/AIDS research in Botswana. International Journal of Qualitative Studies in Education, 18, 659–684.

[daab152-B18] Cialdini R. B. (2007) Descriptive social norms as under-appreciated sources of social control. Psychometrika, 72, 263–268.

[daab152-B19] Corbin J. H. , Mittelmark M. B. (2008) Partnership lessons from the Global Programme for Health Promotion Effectiveness: a case study. Health Promotion International, 23, 365–371. 18835888 10.1093/heapro/dan029

[daab152-B20] Corbin J. H. , Sanmartino M., Urke H. B., Hennessy E. A. (2021) Arts, health promotion, and social justice: Synergy in motion. In Corbin J. H., Sanmartino M., Hennessy E. A. and Urke H. B. (eds), Arts and health promotion: tools and bridges for practice, research, and social transformation. Springer International Publishing, New York, NY, pp. 345–356. 10.1007/978-3-030-56417-9_21.

[daab152-B21] Cuan-Baltazar J. Y. , Muñoz-Perez M. J., Robledo-Vega C., Pérez-Zepeda M. F., Soto-Vega E. (2020) Misinformation of COVID-19 on the internet: infodemiology study. JMIR Public Health and Surveillance, 6, e18444. 32250960 10.2196/18444 PMC7147328

[daab152-B22] Datta S. , Mullainathan S. (2012) Behavioral design: a new approach to development policy. Center for Global Development, Policy Paper, 016. http://www.cgdev.org/content/publications/detail/1426679 (last accessed 21 May 2021).

[daab152-B23] De Weger E. , Van Vooren N. J. E., Drewes H. W., Luijkx K. G., Baan C. A. (2020) Searching for a new community engagement approach in the Netherlands: a realist qualitative study. BMC Public Health, 20, 508. 32299398 10.1186/s12889-020-08616-6 PMC7164336

[daab152-B24] Dixon B. E. , Caine V. A., Halverson P. K. (2020) Deficient response to COVID-19 makes the case for evolving the public health system. American Journal of Preventive Medicine, 59, 887–891. 32978011 10.1016/j.amepre.2020.07.024 PMC7448961

[daab152-B25] Ekzayez A. , al-Khalil M., Jasiem M., Al Saleh R., Alzoubi Z., Meagher K. et al (2020) COVID-19 response in northwest Syria: innovation and community engagement in a complex conflict. Journal of Public Health (Oxford, England), 42, 504–509. 10.1093/pubmed/fdaa068 PMC7313796 32436578

[daab152-B26] Elisala N. , Turagabeci A., Mohammadnezhad M., Mangum T. (2020) Exploring persons with disabilities preparedness, perceptions and experiences of disasters in Tuvalu. PLOS One, 15, e0241180. 33119660 10.1371/journal.pone.0241180 PMC7595426

[daab152-B27] Gilmore B. , Ndejjo R., Tchetchia A., de Claro V., Mago E., Diallo A. A. et al (2020) Community engagement for COVID-19 prevention and control: a rapid evidence synthesis. BMJ Global Health, 5, e003188. 10.1136/bmjgh-2020-003188 PMC7554411 33051285

[daab152-B28] Glanz K. , Rimer B. K., Viswanath K. (2008) Health behavior and health education, 4th edition. John Wiley & Sons, San Francisco, CA.

[daab152-B29] Gonah L. (2020) Key considerations for successful risk communication and community engagement (RCCE) programmes during COVID-19 pandemic and other public health emergencies. Annals of Global Health, 86, 146. 33262935 10.5334/aogh.3119 PMC7678561

[daab152-B30] Haider H. (2017) Changing gender and social norms, attitudes and behaviours. K4D Helpdesk Research Report series, Institute of Development Studies, Brighton, UK. https://gsdrc.org/publications/changing-gender-social-norms-attitudes-behaviours/ (last accessed 21 May 2021).

[daab152-B31] Hu G. , Qiu W. (2020) From guidance to practice: promoting risk communication and community engagement for prevention and control of coronavirus disease (COVID-19) outbreak in China. Journal of Evidence-Based Medicine, 13, 168–172. 32445287 10.1111/jebm.12387 PMC7280730

[daab152-B32] Institute of Development Studies (IDS). (2015) Local engagement in Ebola outbreaks and beyond in Sierra Leone. https://core.ac.uk/download/pdf/237086117.pdf (last accessed 21 May 2021).

[daab152-B33] International Federation of Red Cross (IFRC). (2019) From words to action: Towards a community-centred approach to preparedness and response in health emergencies. https://apps.who.int/gpmb/assets/thematic_papers/tr-5.pdf (last accessed 21 May 2021).

[daab152-B34] International Union for Health Promotion and Education (IUHPE). (2021) COVID-19 response for the African region. https://www.iuhpe.org/index.php/en/health-promotion-systems/covid-19-response-for-the-african-region (last accessed 21 May 2021).

[daab152-B35] Kenya National Bureau of Statistics (2019). Kenya population and housing census volume II: distribution of population by administrative units, 21-26 February. https://www.knbs.or.ke/?wpdmpro=2019-kenya-population-and-housing-census-volume-ii-distribution-of-population-by-administrative-units (last accessed 21 May 2021).

[daab152-B36] Laverack G. (2017) The role of health promotion in disease outbreaks and health emergencies. Societies, 7, 2.

[daab152-B37] Laverack G. , Manoncourt E. (2016) Key experiences of community engagement and social mobilization in the Ebola response. Global Health Promotion, 23, 79–82. 26518037 10.1177/1757975915606674

[daab152-B38] Leach M. , MacGregor H., Scoones I., Wilkinson A. (2021) Post-pandemic transformations: how and why COVID-19 requires us to rethink development. World Development, 138, 105233. 33100478 10.1016/j.worlddev.2020.105233 PMC7566764

[daab152-B39] Leitch S. , Corbin J. H., Boston-Fisher N., Ayele C., Delobelle P., Gwanzura Ottemöller F. et al (2021) Black Lives Matter in health promotion: moving from unspoken to outspoken. Health Promotion International, 36, 1160–1169. 33305322 10.1093/heapro/daaa121 PMC7953963

[daab152-B40] Levin-Zamir D. , Sørensen K., Su T. T., Sentell T., Rowlands G., Messer M. et al (2021) Health promotion preparedness for health crises – a ‘must’ or ‘nice to have’? Case studies and global lessons learned from the COVID-19 Pandemic. Global Health Promotion, 28, 27–37. 33775167 10.1177/1757975921998639 PMC8246413

[daab152-B41] Lhussier M. , Lowe N., Westaway E., Dykes F., Mckeown M., Munir A. et al (2016) Understanding communication pathways to foster community engagement for health improvement in North West Pakistan. BMC Public Health, 16, 591. 27430317 10.1186/s12889-016-3222-7 PMC4950241

[daab152-B42] Limaye R. J. , Sauer M., Ali J., Bernstein J., Wahl B., Barnhill A. et al (2020) Building trust while influencing online COVID-19 content in the social media world. The Lancet. Digital Health, 2, E277–E278. 32322814 10.1016/S2589-7500(20)30084-4 PMC7173823

[daab152-B43] Maher R. , Murphet B. (2020) Community engagement in Australia’s COVID-19 communications response: learning lessons from the humanitarian sector. Media International Australia, 177, 113–118.

[daab152-B44] Miller J. (2007) Medical apartheid: the dark history of medical experimentation on Black Americans from colonial times to the present. Psychiatric Services, 58, 1380–1381.

[daab152-B45] Mosby I. , Swidrovich J. (2021) Medical experimentation and the roots of COVID-19 vaccine hesitancy among Indigenous Peoples in Canada. CMAJ, 193, E381–E383. 33627413 10.1503/cmaj.210112 PMC8096406

[daab152-B46] Omoleke S. A. , Mohammed I., Saidu Y. (2016) Ebola viral disease in West Africa: a threat to global health, economy and political stability. Journal of Public Health in Africa, 7, 534. 28299152 10.4081/jphia.2016.534 PMC5349256

[daab152-B47] Patel P. , Meagher K., El Achi N., Ekzayez A., Sullivan R., Bowsher G. (2020) “Having more women humanitarian leaders will help transform the humanitarian system”: challenges and opportunities for women leaders in conflict and humanitarian health. Conflict and Health, 14, 84. 33292351 10.1186/s13031-020-00330-9 PMC7709302

[daab152-B48] Ramirez-Valles J. , Breton E., Chae D. H., Haardörfer R., Kuhns L. M. (2020) The COVID-19 pandemic: everything old is new again in public health education. Health Education & Behavior, 47, 501–503. 32517520 10.1177/1090198120935067

[daab152-B49] RCCE Collective Service. (2021) Survey & Dataset. https://www.rcce-collective.net/data/survey-dataset/ (last accessed 21 May 2021).

[daab152-B50] Romer D. , Jamieson K. H. (2020) Conspiracy theories as barriers to controlling the spread of COVID-19 in the US. Social Science & Medicine, 263, 113356. 32967786 10.1016/j.socscimed.2020.113356 PMC7502362

[daab152-B51] Saboga-Nunes L. , Levin-Zamir D., Bittlingmayer U., Contu P., Pinheiro P., Ivassenko V. et al, (2020). A health promotion focus on COVID-19: keep the Trojan horse out of our health systems: promote health for all in times of crisis and beyond! International Union for Health Promotion and Education (IUHPE), Montreal. https://eupha.org/repository/sections/hp/A_Health_Promotion_Focus_on_COVID-19_with_S.pdf (last accessed 21 May 2021).

[daab152-B52] Sentell T. , Vamos S., Okan O. (2020) Interdisciplinary perspectives on health literacy research around the world: more important than ever in a time of COVID-19. International Journal of Environmental Research and Public Health, 17, 3010. 10.3390/ijerph17093010 PMC7246523 32357457

[daab152-B53] Sherpa D. (2020) Estimating impact of austerity policies in COVID-19 fatality rates: examining the dynamics of economic policy and case fatality rates (CFR) of COVID-19 in OECD countries. MedRxiv, April 13, 2020: 10.1101/2020.04.03.20047530.

[daab152-B54] Shrivastava S. R. , Shrivastava P. S. (2020) Short communication: strengthening risk communication and community engagement for the containment of the Corona Virus Disease 2019 outbreak. African Health Sciences, 20, 1164–1165. 33402961 10.4314/ahs.v20i3.18 PMC7751521

[daab152-B55] Smith J. A. , Judd J. (2020) COVID-19: vulnerability and the power of privilege in a pandemic. Health Promotion Journal of Australia, 31, 158–153. 32197274 10.1002/hpja.333 PMC7165578

[daab152-B56] Sørensen K. (2020) Lack of alignment in emergency response by systems and the public: a Dutch disaster health literacy case study. Disaster Medicine and Public Health Preparedness, 1–4. 10.1017/dmp.2020.226. 32958081

[daab152-B57] Tam W. J. (2021) A national risk communication and community engagement campaign for large, closed communities in Singapore. Collective Service, 12 April. https://www.rcce-collective.net/case-study/a-national-risk-communication-and-community-engagement-campaign-for-large-closed-communities-in-singapore/ (last accessed 21 May 2021).

[daab152-B58] Tasnim S. , Hossain M. M., Mazumder H. (2020) Impact of rumors and misinformation on COVID-19 in social media. Journal of Preventive Medicine and Public Health, 53, 171–174. 32498140 10.3961/jpmph.20.094 PMC7280809

[daab152-B59] Tilley H. (2020) COVID-19 across Africa: colonial hangovers, racial hierarchies, and medical histories. Journal of West African History, 6, 155–179.

[daab152-B60] Tindana P. O. , De Vries J., Kamuya D. (2020) Ethical challenges in community engagement practices in research during the COVID-19 pandemic in Africa. AAS Open Research, 3, 23.

[daab152-B61] Toppenberg-Pejcic D. , Noyes J., Allen T., Alexander N., Vanderford M., Gamhewage G. (2019) Emergency risk communication: lessons learned from a rapid review of recent gray literature on Ebola, Zika, and Yellow Fever. Health Communication, 34, 437–455. 29558199 10.1080/10410236.2017.1405488

[daab152-B62] United Nations (UN). (2011) Communication for development: strengthening the effectiveness of the United Nations. http://www.unicef.org/cbsc/files/Inter-agency_C4D_Book_2011.pdf (last accessed 21 May 2021).

[daab152-B63] United Nations (UN) OCHA (2016). Coordinated community engagement humanitarian action experiences in Asia: workshop report. Bangkok, Thailand, 4-5 October. https://reliefweb.int/sites/reliefweb.int/files/resources/Workshop%20Report%20Final_Community%20Engagement%20in%20Humanitarian%20Action.pdf (last accessed 21 May 2021).

[daab152-B64] Uscinski J. E. , Enders A. M., Klofstad C., Seelig M., Funchion J., Everett C. et al (2020) Why do people believe COVID-19 conspiracy theories? Harvard Kennedy School Misinformation Review, 1, 1–12. 10.37016/mr-2020-38 PMC8345314 34368805

[daab152-B65] Van den Broucke S. (2020) Why health promotion matters to the COVID-19 pandemic, and vice versa. Health Promotion International, 35, 181–186. 32297931 10.1093/heapro/daaa042 PMC7184433

[daab152-B66] Wang M. , Han X., Fang H., Xu C., Lin X., Xia S. et al (2018) Impact of health education on knowledge and behaviors toward infectious diseases among students in Gansu Province, China. BioMed Research International, 2018, 1. 10.1155/2018/6397340 PMC5863350 29707573

[daab152-B67] Waisbord S. (2014) Where do we go next? Behavioral and social change for child survival. Journal of Health Communication, 19 (Suppl 1), 216–222. 25207454 10.1080/10810730.2014.933288 PMC4205915

[daab152-B68] Wieland M. L. , Asiedu G. B., Lantz K., Abbenyi A., Njeru J. W., Osman A., Rochester Healthy Community Partnership COVID-19 Task Force. et al (2020) Leveraging community engaged research partnerships for crisis and emergency risk communication to vulnerable populations in the COVID-19 pandemic. Journal of Clinical and Translational Science, 5, e6. 33942018 10.1017/cts.2020.47 PMC7605400

[daab152-B69] World Health Organization (WHO). (2005) International Health Regulations (2005) Third Edition. World Health Organization. https://www.who.int/publications-detail-redirect/9789241580496 (last accessed 24 August 2021).

[daab152-B70] World Health Organization (WHO). (1986) WHO The Ottawa Charter for Health Promotion. http://www.who.int/healthpromotion/conferences/previous/ottawa/en/ (last accessed 21 May 2021).

[daab152-B71] World Health Organization (WHO). (2017) WHO community engagement framework for quality, people-centred and resilient health services. https://apps.who.int/iris/bitstream/handle/10665/259280/WHO-HIS-SDS-2017.15-eng.pdf;jsessionid=AA6DC354837011B232FC4AE77982395E?sequence=1 (last accessed 21 May 2021).

[daab152-B72] World Health Organization (WHO). (2018) Thirteenth general programme of work 2019-2023. https://apps.who.int/iris/bitstream/handle/10665/324775/WHO-PRP-18.1-eng.pdf (last accessed 21 May 2021).

[daab152-B73] World Health Organization (WHO). (2019) Health emergency and disaster risk management framework. https://www.who.int/hac/techguidance/preparedness/health-emergency-and-disaster-risk-management-framework-eng.pdf (last accessed 21 May 2021).

[daab152-B74] World Health Organization (WHO). (2020) COVID-19 global risk communication & community engagement strategy, 23 December. https://www.who.int/publications/i/item/covid-19-global-risk-communication-and-community-engagement-strategy (last accessed 21 May 2021).

[daab152-B75] World Health Organization (WHO). (2021) Everyone’s business: whole of society actions to manage health risks and reduce socioeconomic impacts of emergencies and disasters. https://apps.who.int/iris/bitstream/handle/10665/339421/9789240015081-eng.pdf?sequence=1&isAllowed=y (last accessed 21 May 2021).

[daab152-B76] Zhu J. , Cai Y. (2020) Engaging the communities in Wuhan, China during the COVID-19 outbreak. Global Health Research and Policy, 5, 35. 32685692 10.1186/s41256-020-00162-3 PMC7356108

